# Feasibility of mobilisation in ICU: a multi-centre point prevalence study of mobility practices in the UK

**DOI:** 10.1186/s13054-023-04508-4

**Published:** 2023-06-01

**Authors:** Claire Black, Helen Sanger, Ceri Battle, Allaina Eden, Evelyn Corner

**Affiliations:** 1 University College London Hospitals NHS Foundation Trust, London, NW1 2BU UK; 2 Newcastle upon Tyne Hospitals NHS Foundation Trust, Queen Victoria Road, Newcastle upon Tyne, NE1 4LP UK; 3 Physiotherapy Dept, Morriston Hospital, Swansea, SA6 6NL UK; 4 Royal Papworth Hospital, Papworth Road, Cambridge, CB2 0AY UK; 5 33n Ltd., 9 Quy Court, Colliers Lane, Stow-Cum-Quy, Cambridge, Cambridgeshire CB25 9AU England, UK

**Keywords:** Critical illness, Critical care, Mobilisation, Rehabilitation, Intensive care unit

## Abstract

**Background:**

Early mobilisation in critical care is recommended within clinical guidance; however, mobilisation prevalence across the UK is unknown. The study aimed to determine the proportion of patients mobilised out of bed within 48–72 h, to describe their physiological status, and to compare this to published consensus safety recommendations for out-of-bed activity.

**Methods:**

A UK cross-sectional, multi-centre, observational study of adult critical care mobility practices was conducted. Demographic, physiological and organ support data, mobility level, and rationale for not mobilising out of bed, were collected for all patients on 3rd March 2022. Patients were categorised as: Group 1—mobilised ICU Mobility Scale (IMS) ≥ 3; Group 2—not-mobilised IMS < 3 with physiological reasons; or Group 3—not-mobilised IMS < 3 with non-physiological barriers to mobilisation. Rationale for the decision to not mobilise was collected qualitatively. Regression analysis was used to compare the physiological parameters of Group 1 (mobilised) versus Group 2 (not-mobilised with physiological reasons). Patients were stratified as ‘low-risk’, ‘potential-risk’ or ‘high-risk’ using published risk of adverse event ratings.

**Results:**

Data were collected for 960 patients across 84 UK critical care units. Of these 393 (41%) mobilised, 416 (43%) were not-mobilised due to physiological reasons and 151 (16%) were not mobilised with non-physiological reasons. A total of 371 patients had been admitted for ≤ 3 days, of whom 180 (48%) were mobilised, 140 (38%) were not mobilised with physiological reasons, and 51 (14%) were not mobilised with non-physiological reasons. Of the 809 without non-physiological barriers to mobilisation, 367 (45%) had a low risk of adverse event rating and 120 (15%) a potential risk, of whom 309 (84%) and 78 (65%) mobilised, respectively. Mobility was associated with a Richmond Agitation-Sedation Scale of − 1 to + 1, lower doses of vasoactive agents, a lower inspired oxygen requirement.

**Conclusion:**

Although only 40% of patients mobilised out of bed, 89% of those defined ‘low-risk’ did so. There is significant overlap in physiological parameters for mobilisation versus non-mobilisation groups, suggesting a comprehensive physiological assessment is vital in decision making rather than relying on arbitrary time points.

*Clinical Trials registration***:** NCT05281705 Registered March 16, 2022. Retrospectively registered.

**Supplementary Information:**

The online version contains supplementary material available at 10.1186/s13054-023-04508-4.

## Introduction

The anabolic and cardiovascular benefits of exercise are well recognised in both health and many chronic disease states [1]. Early studies of physical activity in critical illness designed to mitigate the impact of Intensive Care Unit-acquired weakness (ICUAW) [2–4] have demonstrated improved function. This evidence contributed to national UK guidance recommending instigation of early mobilisation within the critical care setting [5].

Nonetheless, over the following decade multiple controlled trials have failed to demonstrate superiority of any specific type, dose, or intensity of rehabilitation within critical care, with the majority showing little or no difference between the intervention and usual care [6–8]. It has been argued that the benefits of mobilisation were only shown in those trials whose intervention commenced within 72 h of critical care admission [2, 9–11]. Combined with a growing appreciation of the rapidity of onset of ICUAW, this has led to a particular focus on ‘early mobility’ in critical illness [4, 12, 13].

A key challenge to the implementation of early mobility is patient safety. Only a small percentage of critical care patients meet physiological inclusion criteria for many rehabilitation trials, raising the question of generalisability of results. An international expert consensus on the safety of mobilising mechanically ventilated adults outlined suggested parameters of physiological status within which active mobility is safe [14]. The authors specifically commented that an undue focus on potential adverse events attributed to mobilisation may result in missed opportunities to demonstrate benefit. However, point prevalence studies continue to show relatively low overall rates of active mobility [15–19].

A further barrier to the evidence base for mobilisation of critical care patients is the lack of definition of ‘usual care’ received by control patients in almost all studies. In the UK, this is assumed to be the provision of some physical rehabilitation, with timing, dose and type of rehabilitation determined by clinician assessment, as recommended by the National Institute for Health and Care Excellence [5]. There are no published studies of usual practice within UK critical care units against which mobilisation strategies can be compared or whether those undertaking out-of-bed mobilisation satisfy safety criteria. In addition, the proportion of patients who do so within the first 48–72 h is unknown.

The aim of this study was to describe the mobility practices in adult critical care units across the UK, to provide a snapshot of usual care. Specifically,

(i) to determine the level of mobility achieved by patients in UK critical care units on a given day;
(ii) to determine the physiological profile of patients that did, and did not mobilise out of bed;
(iii) to compare these physiological profiles with published international safety parameters for mobilisation out of bed;
(iv) to determine the proportion of patients that mobilised out of bed within the first 72 h of admission.

## Methods

This was a cross-sectional, multi-centre, observational study of adult critical care mobility practice across the UK on a single day in March 2022. The study was approved by the UK National Research Ethics Service (REC reference number 20/LO/0061) who waived the need for patient or next-of-kin consent. The study was carried out in 36 NHS organisations. All patients present in level 2 (i.e. requiring more detailed observation or intervention, including support of single organ dysfunction or post-operative care) and level 3 (i.e. needing advanced respiratory support alone, or requiring basic respiratory support plus support of at least two organ systems**)** critical care areas in each organisation on the 3rd March 2022 at 00:00 were included. There were no exclusion criteria. Study data were entered into the REDCap electronic data capture tools hosted at University College London [20] by either physiotherapists and or research nurses at each site. All data collectors had received prior training on completion of the survey form. Demographic, physiological and organ support data were collected (Additional file 1: Table S1) from either the patients electronic health record or paper chart. Reasons for a clinical decision to not mobilise the patient out of bed were collated qualitatively via the open question: ‘give reasons why a greater level of mobility was not achieved’. All data were pseudonymised.

Patient mobility level was measured by the ICU Mobility Scale (IMS), an 11-point ordinal scale of ‘highest level of mobility’ achieved on the day. An IMS of 0 is passively lying in bed, and IMS 10 is independently mobilising five metres or more without an aid [21]. Out-of-bed mobilisation was defined as per Hodgson and colleagues’ international consensus paper, as “any activity where the patient sits over the edge of the bed (dangling), stands, walks, marches on the spot or sits out of bed” [14]. That is, an IMS of three or more.

A vasoactive-inotropic score (VIS) [22] was used to standardise vasoactive drug doses for each patient (Additional file 1: Table S5). As VIS does not include the use of the vasopressor, metaraminol, this was compared separately between groups.

Post hoc a risk of an adverse event rating, red—high risk, amber—potential risk or green—low risk as described by Hodgson et al. [14], was calculated for each patient using the parameters available within the data set (Additional file 1: Table S6). A single parameter in the red (high risk of an adverse event) category categorises the patient as red—high risk to mobilise. To be green, all parameters would need to fall in the low-risk category.

## Analysis.

Patients were allocated to one of three groups; Group 1—mobilised ICU Mobility Scale (IMS) ≥ 3; Group 2—not-mobilised IMS < 3 with physiological reasons for not mobilising; or Group 3—not-mobilised IMS < 3 with non-physiological barriers to mobilisation. The non-physiological reasons for not mobilising are given in Table 1.

**Table 1:** Non-physiological barriers to mobilisation

Reason for not mobilising out of bed	*n*
Movement restrictions—appropriate mobility level achieved	38
Staffing	30
Awaiting medical procedure	22
Dying	16
Intervention as per site rehabilitation plan	14
Patient declined	12
At baseline level of mobility	8
Medical procedure in progress	8
Motor block	2
Equipment	1

All variables were inspected for missingness (Additional file 1: Table S4). Continuous variables are reported as median and interquartile range (IQR) or mean and standard deviation (SD) depending on the test for normality which was assessed by the Shapiro–Wilk test. Categorical data were reported as frequency counts and percentages. Where comparisons are made between the 3 groups (mobilised; not mobilised; non-physiological barriers to mobilisation), Fisher’s exact test was used for categorical values and Mann–Whitney *U* test for the continuous variables. Statistical significance was assumed at *p* < 0.05. A Generalised Linear Model with ‘mobilised’ or ‘not-mobilised’ as the explanatory variable was used to explore the combined physiological parameters of patients without non-physiological barriers to mobilisation. A Chi-square test of independence was performed to examine the relationship between the ‘low-risk’ category and mobilising. All data were analysed using R [23].

An alluvial plot was generated to illustrate the risk of adverse events category and whether out-of-bed mobility was achieved.

## Results

Data were recorded for 960 patients across 84 level 2 or 3 critical care areas in 36 NHS organisations. The cohort description is shown in Table 2.

**Table 2:** Characteristics of all patients

	*n* (%)
*Type of admission*	960
Elective surgery	231 (24.1)
Emergency surgery	199 (20.7)
Medical	456 (47.5)
Other	74 (7.7)
Gender = male	606 (63.1)
*Airway*	
Nasal ETT	1 (0.1)
Oral ETT	283 (29.5)
Own	482 (50.2)
Tracheostomy	194 (20.2)
*Advanced support*	
Nova lung	3 (0.3)
ECMO	10 (1.0)
RRT	100 (10.4)
*Cardiac assist device*	
BiVAD	5 (0.5)
IABP	7 (0.7)
LVAD	1 (0.1)
RVAD	2 (0.2)
TAH	1 (0.1)

ETT, endotracheal tube; ECMO, extracorporeal membrane oxygenation; RRT, renal replacement therapy; BiVAD, biventricular assist device; IABP, intra-aortic balloon pump; LVAD, left ventricular assist device; RVAD, right ventricular assist device; TAH, total artificial heart

**Fig. 1:**
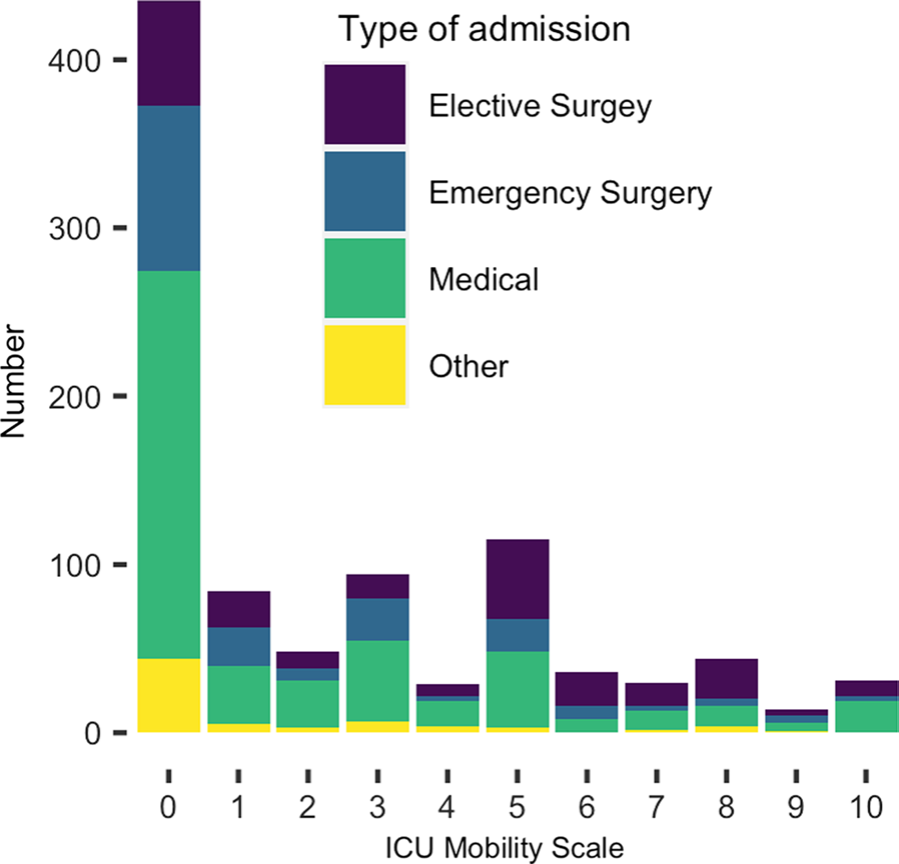
ICU Mobility Scale (all patients) on the study day

**Fig. 2:**
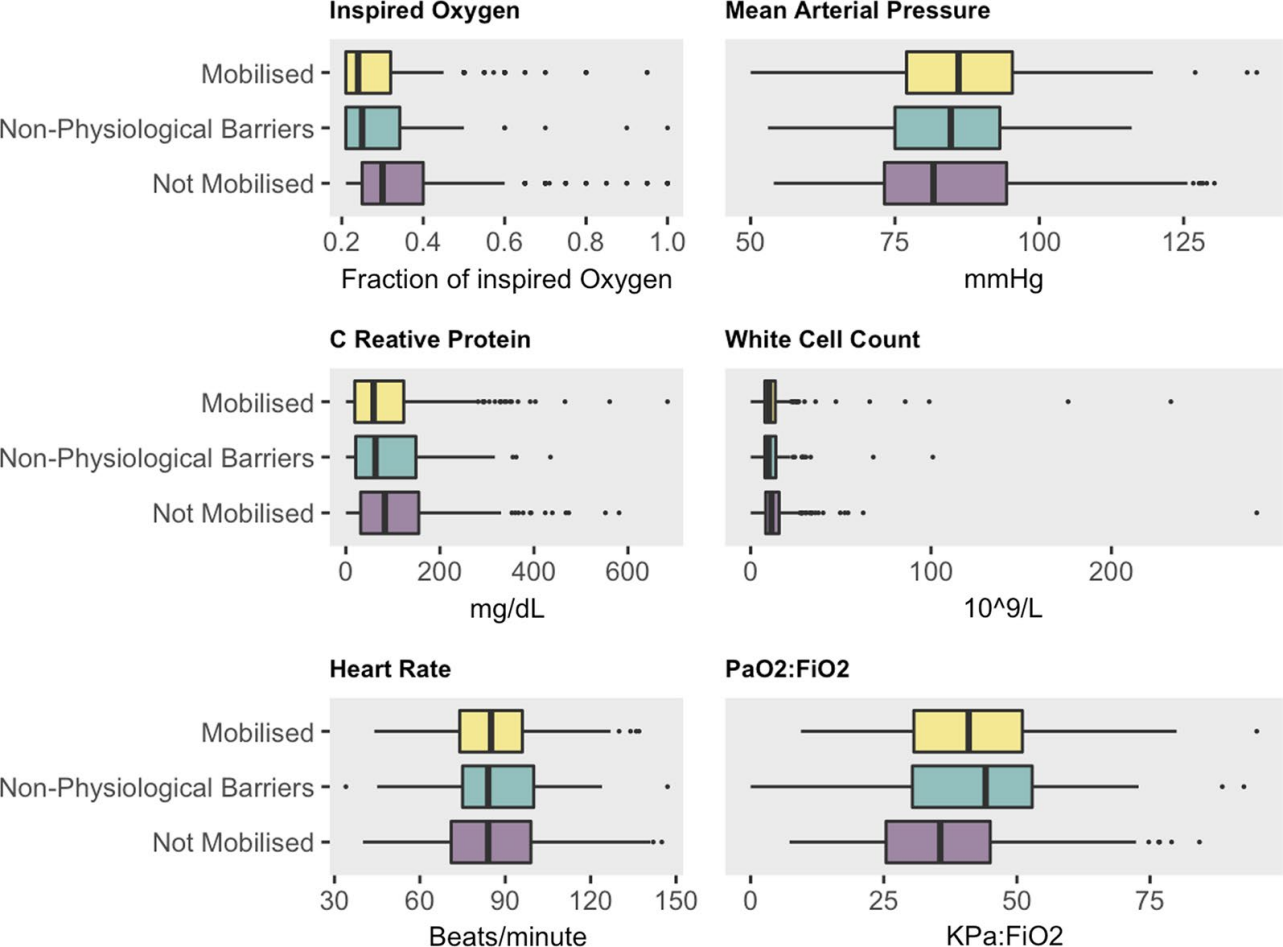
Parameters with statistically different values between mobilised, not-mobilised and non-physiological barriers to mobilisation

393 (41%) of patients were mobilised on the day of the study, 416 (43%) were not mobilised, and 151 (16%) had non-physiologically barriers to mobilisation. Figure 1 shows the IMS achieved for all patients. The characteristics of the 3 groups (mobilised, not mobilised, non-physiologically barriers to mobilisation) are given in Table 3, and plots of the statistically significant variables in Fig. 2. P:F (Kpa:FiO_2_) was only calculatable for 753 patients as either PaO_2_ or FiO_2_ was not reported for 207 patients.

**Table 3:** Characteristics of all patients by mobility status

	Mobilised	Not mobilised	Non-physiological barriers	*p*
*N*	393	416	151	
*Type of admission* (%)				< 0.001
Elective surgery	139 (35.4)	69 (16.6)	23 (15.2)	
Emergency surgery	70 (17.8)	93 (22.4)	36 (3.8)	
Medical	163 (41.5)	225 (54.1)	68 (45.0)	
Other	21 (5.3)	29 (6.9)	24 (15.9)	
Gender = male (%)	251 (63.9)	255 (61.3)	100 (66.2)	0.519
RRT (%)	17 (4.3)	70 (16.8)	13 (8.6)	< 0.001
*Airway*				< 0.001
Nasal ETT	0 (0.0)	1 (0.2)	0 (0.0)	
Oral ETT	9 ( 2.3)	236 (56.7)	38 (25.2)	
Own	307 (78.1)	105 (25.2)	70 (46.4)	
Tracheostomy	77 (19.6)	74 (17.8)	43 (28.5)	
Nova lung (%)	1 (0.3)	2 (0.5)	0 (0.0)	1
ECMO (%)	2 (0.5)	4 (1.0)	4 (2.6)	0.113
*Cardiac assist device* (%)				0.054
BiVAD	3 (0.8)	2 (0.5)	0 (0.0)	
IABP	0 (0.0)	7 (1.7)	0 (0.0)	
LVAD	1 (0.3)	0 (0.0)	0 (0.0)	
RVAD	2 (0.5)	0 ( 0.0)	0 (0.0)	
TAH	1 (0.3)	0 (0.0)	0 (0.0)	
FiO_2_ (median [IQR])	0.24 [0.21, 0.32]	0.30 [0.25, 0.40]	0.25 [0.21, 0.34]	< 0.001
PF (median [IQR])	40.94 [30.64, 51.03]	35.68 [25.49, 45.10]	44.12 [30.37, 52.87]	< 0.001
Haemoglobin (mean (SD))	97.21 (23.83)	93.93 (21.02)	92.08 (23.73)	0.028
CRP (median [IQR])	59.00 [19.00, 123.08]	83.00 [31.50, 155.05]	63.00 [21.00, 149.00]	0.008
WCC (median [IQR])	10.30 [7.90, 13.70]	11.50 [8.33, 15.80]	9.93 [7.90, 14.00]	0.001
MAP (mean (SD))	86.83 (14.14)	84.17 (15.90)	85.16 (13.78)	0.039
HR (mean (SD))	85.25 (17.55)	86.45 (20.14)	86.22 (18.29)	0.651
VIS total (median [IQR])	0.00 [0.00, 0.00]	0.00 [0.00, 4.00]	0.00 [0.00, 0.00]	< 0.001
Metaraminol dose (mean (SD))	1.09 (0.56)	0.99 (0.53)	1.01 (0.51)	0.847

ETT, endotracheal tube; ECMO, RRT, renal replacement therapy, extracorporeal membrane oxygenation; BiVAD, biventricular assist device; IABP, intra-aortic balloon pump; LVAD, left ventricular assist device; RVAD right ventricular assist device; TAH, total artificial heart, FiO_2_, fraction of inspired oxygen, PF, PaO_2_ (Kpa) fraction of inspired oxygen ratio, CRP, C reactive protein, WCC,  white cell count, MAP, mean arterial pressure, HR, heart rate, VIS, vasoactive-inotropic score

Of the 960 patients, 371 (39%) had been admitted for ≤ 3 days. Of those, 180 (48%) were mobilised, 140 (38%) were not mobilised, and 51 (14%) had non-physiologically barriers to mobilisation. Characteristics of the three groups are given in Additional file 1: Table S3.

Within the cohort without non-physiological barriers to mobilisation, the odds of mobilising if sedated with a RASS score outside the range − 1 to + 1 was 0.0641, decreasing by 0.93 for every point increase in VIS, and by 0.12 for every point increase in FiO_2_ (Table 4).

**Table 4:** Regression model

	Estimate	Odds ratio	Std. error	*z* value	Pr(> |*z*|)
(Intercept)	− 1.3496	0.2593	0.4918	− 2.7441	0.0061
${\text{VIS}}\;{\text{total}}$	− 0.0726	0.9300	0.026	− 2.7957	0.0052
${\text{RASS}} \ge - 1 \le + 1$	2.6859	14.6715	0.448	5.9957	0
${\text{RASS}} \le - 2 \ge + 2$	− 2.748	0.0641	1.0937	− 2.5126	0.012
${\text{FiO}}2$	− 2.0814	0.1248	0.7434	− 2.7999	0.0051

VIS, vasoactive-inotropic score; RASS, Richmond Agitation-Sedation Score

**Fig. 3:**
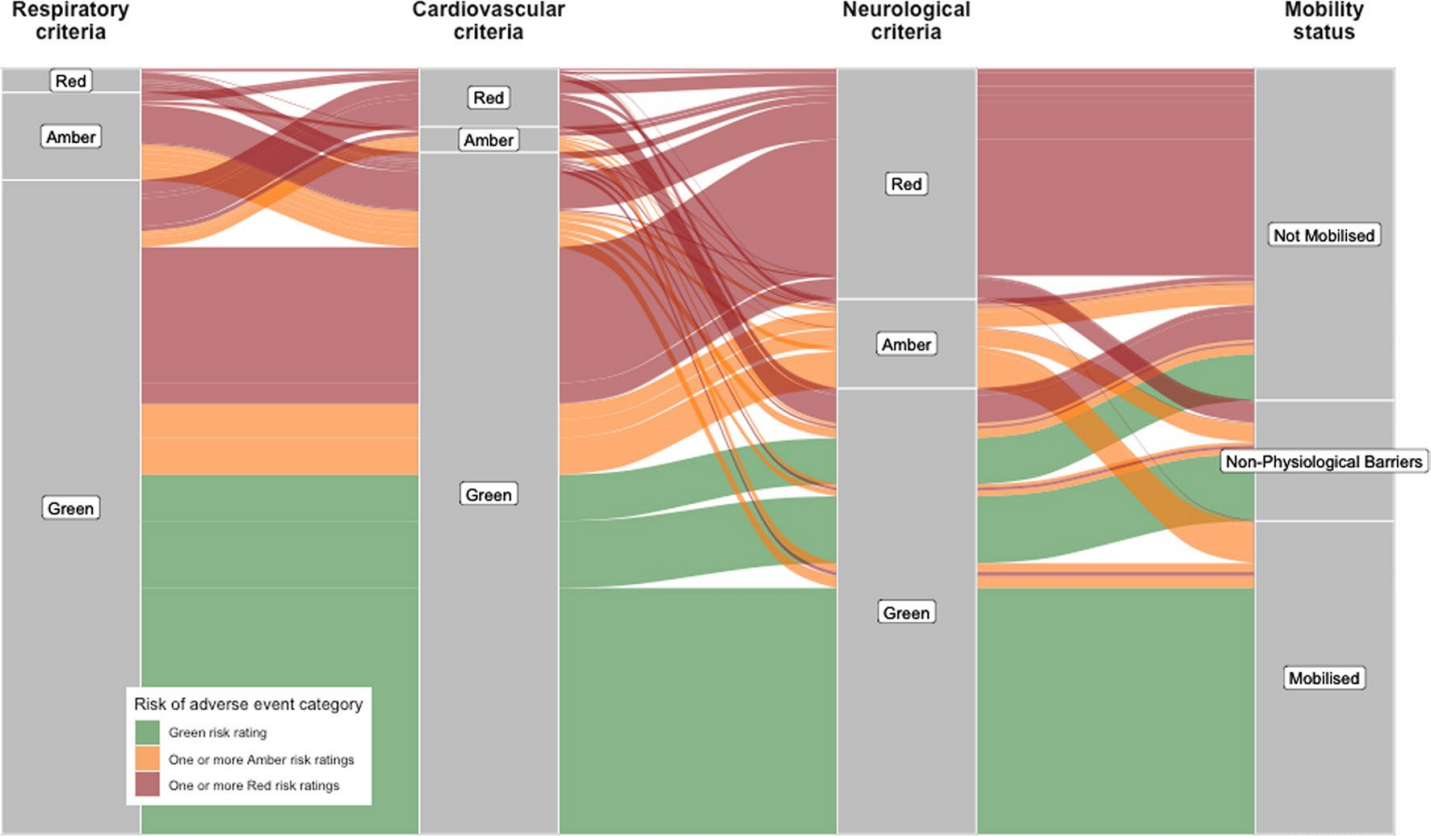
Alluvial plot to illustrate the calculated risk rating for each organ system and the mobility status

Of the 809 patients without non-physiological barriers to mobilisation, 367 (45%) had a green rating and 120 (15%) had an amber rating, of whom 309 (84%) and 78 (65%) mobilised, respectively (Table 5). The alluvial plot in Fig. 3 shows the distribution of the risk of adverse event ratings and organ systems to which the score is attributable. Table 6 summarises reasons why amber and green risk patients were not mobilised out of bed.

**Table 5:** Patients without non-physiological barriers to mobilisation and associated risk of adverse event rating for (all patients)

Risk of adverse event rating	Mobilised	Not mobilised
Green	309 (84%)	58 (16%)
Amber	78 (65%)	42 (35%)
Red	6 (2%)	316 (98%)

**Table 6:** Reasons for not mobilising out of bed in patients with amber or green risk ratings

Reason for not mobilising	Amber	Green
Imminent extubation	4	6
Fatigue	2	8
Uncodable	3	5
Vomiting	1	5
Pain	4	12
Delirium	4	1
Intubated and ventilated	4	2
Perceived low physiological reserve	2	4
Airway clearance essential	1	NA
Unstable-nonspecific	3	1
Unable to engage in activity	4	2
Renal replacement therapy	3	NA

Of the 320 patients in critical care ≤ 3 days without non-physiological barriers to mobilisation, 173 (54%) had a green risk rating and 28 (9%) an amber risk rating, of whom 155 (90%) and 21 (75%) mobilised, respectively (Table 7). Of the 180 patients who mobilised, 109 (60%) were elective surgery patients. The risk ratings for the 172 non-elective admissions were 86 (50%) red: 19 (11%) amber and 67 (39%) green (Table 8). Of the green and amber non-elective surgery admissions, 56 (84%) and 13 (68%) mobilised, respectively. The relationship between the low-risk category and mobilising was significant, *X*^2^ (1, *N* = 809) = 338.54, *p* value < 0.001.

**Table 7:** Out-of-bed mobilisation risk ratings for patients admitted to critical care ≤ 3 days

Risk rating	Mobilised	Not mobilised
Green	155 (89.60%)	18 (10.40%)
Amber	21 (75.00%)	7 (25.00%)
Red	4 (3.36%)	115 (96.64%)

**Table 8:** Out-of-bed mobilisation risk ratings for non-elective surgery patients admitted to critical care ≤ 3 days

Risk rating	Mobilised	Not mobilised
Green	56 (84%)	11 (16%)
Amber	13 (68%)	6 (32%)
Red	2 (2%)	84 (98%)

**Fig. 4:**
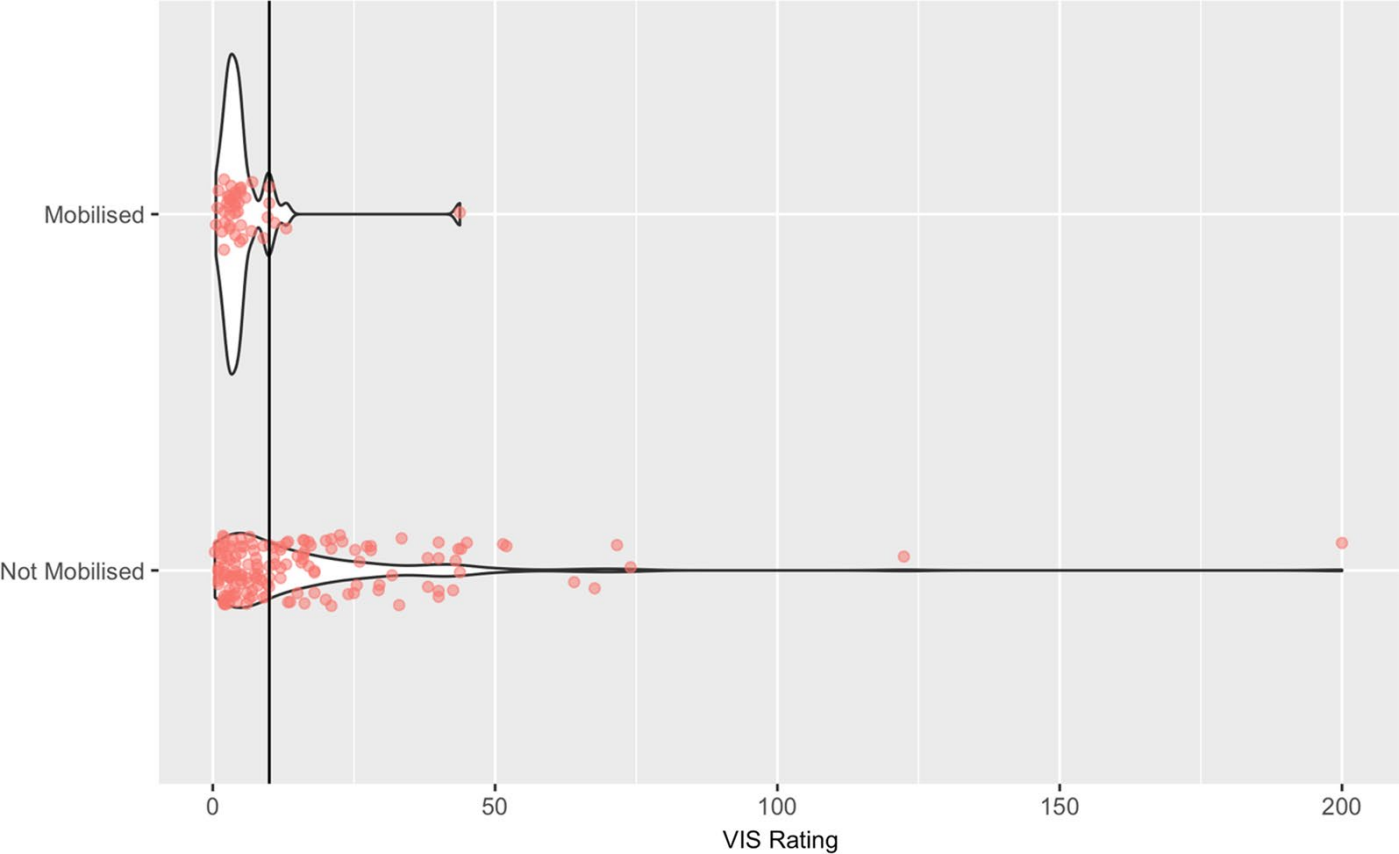
VIS vasoactive-inotropic score (VIS) rating and whether out-of-bed mobility was achieved

There was clear separation of the VIS between patients who were and were not mobilised. The VIS distribution is shown in Fig. 4. There was no difference in the dose of metaraminol between the mobilised and non-mobilised patients (sample estimate 0.08, 95% confidence Interval (− 0.28, 0.58)).

## Discussion

To our knowledge, this is the largest point prevalence study of mobilisation practices in critical care outside of the COVID-19 pandemic. Forty percent of patients mobilised out of bed, higher than the 24–36% range previously published [15–19]. Out-of-bed mobility was associated with a RASS within the range − 1 to + 1, lower doses of vasopressors and inotropes, lower FiO_2_.

While some variables were statistically significant between mobilised and not-mobilised groups, there was considerable overlap of the distribution of these variables. Of note is the lack of clinically meaningful separation of any of the key parameter values.

The majority of patients who met the green risk criteria were mobilised. However, of the those in critical care for less than 72 h, 60% who met the green criteria were elective surgery patients. Only 39% of non-elective patients met green criteria, while 50% fulfilled red criteria. Higher mobility levels in elective surgical patients were also reported in an Australian cohort study [24]. As such, studies focusing solely on elective surgical patients should not be generalised to the broader critical care population. There may be scope for increasing mobility levels in the green non-mobilised group. The Hodgson et al. [14] consensus focused on safe parameters for mechanically ventilated patients. However, the non-mobilised cohort also included those not receiving mechanical ventilatory support. The presence of an artificial airway (whether endotracheal tube or tracheostomy) is classified as a ‘green’ risk characteristic; however, the recommendations specify taking the most conservatively scored individual parameter as group-membership (i.e. any single amber or red), and specifically comment that their aim is to provide guidance to maximise safe mobilisation [14] For these reasons, and the absence of other high-quality guidance on safe mobilisation of critical care patients, the risk rating was applied to the whole cohort in this study.

The wide confidence interval around both the VIS and each individual vasoactive drug suggests that clinical decision making is not based solely on an arbitrary dose, but is dependent on individual patient physiology. The observation that the threshold for mobilising was a VIS of 10 should not be interpreted that it is safe to mobilise all patients with scores < 10. There may be a cohort of patients receiving vasoactive support in whom the cumulative risk of other factors precluding mobilisation is deemed acceptable by those taking the decision to mobilise.

Commentators of critical care mobility practices often report low levels of mobility as problematic. Although these data showed that only 40% of all patients were mobilised, 89% and 75% of those that met green and amber risk ratings (i.e. the only published international consensus on safety of out-of-bed activity) mobilised, respectively. Re-interpreting the data in the context of physiological safety paints a different picture, suggesting 90% of stable patients achieved an appropriate mobility goal within 72 h.

Perhaps there is a time during early severe acute illness when mobilising is neither safe nor feasible. Of note, the ‘early mobility' study by Schweickert et al. [2] reported a significantly shorter time to death in the intervention group. Similarly, the randomised control trial by Schaller and colleagues [10] of early goal-directed mobilisation in a surgical critical care unit showed improved physical function in the intervention group, but a doubling in mortality rate compared to the control limb. The reduction in critical care length of stay is likely to have been confounded by the earlier deaths in the intervention group. A recent multi-centre randomised controlled trial of 750 patients showed no benefit of protocolised early mobilisation versus usual care in terms of morbidity, disability or activities of daily living [13] but an increase in adverse events. This raises the important question of the potential iatrogenic impact of early intensive mobilisation in those with critical illness, and a ceiling effect of dose-benefit, as demonstrated in other populations such as acute stroke [25].

There was a marked physiological variability within groups. Those not mobilised had a pattern of marginally poorer physiological status, but no markers alone met criteria of clinical significance. Hence, each patient’s physiological status is unique, and decision-making is necessarily individualised.

We acknowledge that this observational study has several limitations. Adverse events were not specifically collected, but none were recorded narratively as reasons why a greater level of mobility was not achieved. The 960 patients represent only 16% of the potential critical care capacity in the UK and cover only 20% of the acute health and social care NHS organisation in England, Wales and Northern Ireland, but do represent both specialist and non-specialist hospitals. The data collection on the majority of the sites was carried out by physiotherapists who were part of the patients care team, which may have resulted in less capacity to mobilise patients, potentially reflected in the 30 (3%) patients not mobilising due to staffing issues. However, we believe that this cohort study reflects current practice across the UK.

Variation in usual care altering the dose–effect of mobilisation or other physical rehabilitation interventions is often discussed as a limitation of research in this field. From the data collected in the present study, it could be argued that the lack of well-defined usual care in mobilisation, and significant variation within controls patients, limit the generalisability of the research published to date. Future trials need a formal control group with well-defined usual care in order to make meaningful comparisons with interventions of altered type, dose, timing, or intensity of physical rehabilitation.

## Conclusion

Clinical decision making in UK critical care units broadly follows international guidance on mobilising patients. Although only 40% of all patients mobilised out of bed, almost 90% of those defined as ‘low-risk of adverse events’ did so. Out-of-bed mobility was associated with a lower agitation-sedation score, lower doses of vasoactive agents, and lower FiO_2_. The significant variation in mobilisation practices observed raises a question of the validity of trials comparing physical rehabilitation interventions to ‘usual care’ in UK critical care units without controlling for this heterogeneity.

## Supplementary Information

**Additional file 1.** **Table S1**. Demographic and Physiological data collected. **Table S2**. Non-Physiological barriers to mobilisation. **Table S3**. Summary LOS ≤ 3 days. **Table S4**. Missing data. **Table S5**. Vasoactive-inotropic score calculations. **Table S6**. RAG rating categorisation.

## Data Availability

The data set is not available. We did not seek ethic approval for data to be shared publicly.
