# Supplementary material for: Feasibility of mobilisation in ICU: a multi-centre point prevalence study of mobility practices in the UK
Source: Crit Care. 2023 Jun 1;27:217. doi: 10.1186/s13054-023-04508-4 (PMC10233540; doi:10.1186/s13054-023-04508-4)
Supplement: Supplementary file 1 — Additional file 1. Table S1. Demographic and Physiological data collected. Table S2. Non-Physiological barriers to mobilisation. Table S3. Summary LOS ≤ 3 days. Table S4. Missing data. Table S5. Vasoactive-inotropic score calculations. Table S6. RAG rating categorisation. [file 13054_2023_4508_MOESM1_ESM.pdf]

Suplementary Material

Table 1 – Demographic and Physiological data collected

Field
Critical Care Demographics
ICU speciality
Number of (HDU) level 2 beds
Number of (ICU) level 3 beds
Other ICU speciality
Total number of ICU beds
Number of WTE physiotherapists
Patient Demographics
Age
Type of admission
Date of admission to critical care
Source of admission
Length of stay (days) in previous ICU before transfer
Days of mechanical ventilation
Primary diagnosis
Sex
APACHE II
Comorbidities
Weight in Kg
Height in cm
Clinical Frailty Scale.
Respiratory
Airway
Mode of Respiratory Support
Mandatory or back up respiratory rate
PEEP or EPAP
FiO2
O2 flow in l/min (NC, face mask, Swedish Nose, high flow oxygen)
Total respiratory rate
CVS
Cardiac assist device
ECCO2 removal device
ECMO type
Systolic
Diastolic
MAP
HR
Rhythm
Pharmacological CVS Support
Adrenaline
Noradrenaline
Dobutamine
Dopamine
ADH (vasopressin)
Enoximone

(continued)

Field
Isoprenaline
Levosimendan
Phenylephrine
Milrinone
Metaraminol
Neuro
Paralysed with neuromuscular blocking agents
RASS
GCS
Intracranial pressure (ICP) measured?
Intracranial pressure
CAM-ICU
Sedated
Propofol
Midazolam
Ketamine
Dexmedetomidine
Fentanyl (including 'remi' and 'al')
Labs
pH
PaCO2
PaO2
WCC
K+
Hb
CRP
Creatinine
Bilirubin
Platelets
Renal
Is the patient receiving renal replacement therapy
Vascular Catheter Placement
Rehab
Specific movement restrictions
ICU Mobility Scale score achieved today
Time of intervention
Why did this patient not complete active rehabilitation (against gravity) today? Please give your top 3 reasons for not rehabing.
Highest ICU Mobility Scale score achieved during admission
On what day did this patient first participate in active rehabilitation against gravity (ICU Mobility Scale of 3 or more)?
Did you achieve the ICU Mobility Scale outcome that you were anticipating to complete before the session?
Profession of staff making decision to mobilise
Band of staff making decision to mobilise
Number of staff required to mobilise

Table 2 – Non-Physiological barriers to mobilisation

Reason for Exclusion	n
Movement restrictions – appropriate mobility level achieved	31
Staffing	30
Awaiting medical procedure	22
Dying	16
Intervention as per site rehabilitation plan	14
Patient declined	12
At baseline level of mobility	8
Medical procedure in progress	8
Movement restrictions-specific	7
Motor block	2
Equipment	1

Table 3 – Summary LOS <= 3 days

	Mobilised	Non-Physiological Barriers	Not Mobilised	p
n	180	51	140	
Type of admission (%)				<0.001
Elective Surgey	109 (60.6)	16 (31.4)	39 (27.9)	
Emergency Surgery	24 (13.3)	13 (25.5)	30 (21.4)	
Medical	44 (24.4)	19 (37.3)	63 (45.0)	
Other	3 (1.7)	3 (5.9)	8 (5.7)	
Gender = Male (%)	110 (61.1)	27 (52.9)	86 (61.4)	0.530
Renal Replacement Therapy (%)	2 (1.1)	2 (3.9)	18 (12.9)	<0.001
Airway				<0.001
Nasal ETT	0 (0.0)	0 (0.0)	1 (0.7)	
Oral ETT	2 (1.1)	8 (15.7)	78 (55.7)	
Own	175 (97.2)	40 (78.4)	59 (42.1)	
Tracheostomy	3 (1.7)	3 (5.9)	2 (1.4)	
Nova lung (%)	0 (0.0)	0 (0.0)	1 (0.7)	0.515
ecmo_yn = 0 (%)	180 (100.0)	51 (100.0)	140 (100.0)	NA
cardiac_assist_device = None (%)	180 (100.0)	51 (100.0)	136 (97.1)	0.045
FiO2 median [IQR]	0.24 [0.21, 0.33]	0.24 [0.21, 0.38]	0.30 [0.24, 0.40]	<0.001
P:F median [IQR]	40.18 [30.02, 50.83]	44.24 [27.18, 55.23]	35.93 [25.67, 44.71]	0.026
Haemaglobin median [IQR]	106.00 [89.50, 119.00]	96.00 [84.00, 112.00]	98.50 [84.00, 119.00]	0.184
CRP median [IQR]	66.40 [27.50, 167.50]	73.80 [18.88, 187.57]	90.00 [29.90, 192.75]	0.549
WCC median [IQR]	10.90 [8.72, 13.70]	12.12 [8.27, 14.46]	11.75 [8.95, 15.72]	0.139
MAP median [IQR]	85.17 [76.33, 93.67]	83.67 [76.17, 92.50]	79.33 [71.00, 91.42]	0.036
HR median [IQR]	83.00 [70.00, 90.25]	79.00 [68.00, 97.50]	81.50 [69.75, 95.25]	0.967
VIS total Median [IQR]	0.00 [0.00, 0.00]	0.00 [0.00, 0.00]	0.00 [0.00, 8.50]	<0.001
Metaraminol dose median [IQR]	0.70 [0.47, 1.08]	1.39 [0.49, 1.50]	1.07 [0.43, 1.21]	0.620

Table 4 - Missing data

	Number of missing values
PaO2:FiO2	207
PaCO2	201
PaO2	201
pH	199
Respiratory Rate	53
C Reactive Protein	34
Height	23
Weight	18
FiO2	7
Creatinine	7
Bilirubin	7
Heart Rhythm	4
White Cell Count	4
Potassium	4
Platlets	4
Haemaglobin	2
Primary Diagnosis	1
Cam-ICU	1

Table 5 - Vasoactive-inotropic score calculations

Drug Dose Administrated to Achieve a VIS of 10

Drug	Dose Administrated to Achieve a VIS of 10
Dopamine	10 μg/kg/min
Dobutamine	10 μg/kg/min
Enoximone	10 μg/kg/min
Phenylephrine	1 μg/kg/min
Milrinone	1 μg/kg/min
Olprinone	1 μg/kg/min*
	0.4 μg/kg/min†
Levosimendan	0.2 μg/kg/min
Epinephrine	0.1 μg/kg/min
Norepinephrine	0.1 μg/kg/min
Vasopressin	0.001 U/kg/min (0.07 U/min for a 70 kg patient)

Table 6 – RAG rating categorisation

Item	RAG
**Respiratory**	
FiO2 > 0.6	Amber
SaO2 < 0.9	Red
RR >30	Amber
HFOV	Red
PEEP > 10	Amber
Ventilator Dysynchrony *	Amber
**Rescue Therapies**	
Nitric oxide	Not Collected
Prostocyclin	Not Collected
Prone Positioning	Not Collected
**Cardiovascular**	
IV anti-hypertensives for hypertensive emergency *	Red
MAP Symptomatic below target on vasoactive agents *	Red
Known or suspected severe pulmonary hypertension *	Amber
Bradycardia requiring pharmacological treatment	Red
Bradycardia not requiring pharmacological treatment	Amber
Transvenous or epipericardial pacemaker with dependent rhythm	Red
Vent Dy synchrony*	Amber
ST > 150	Red
ST 120 – 150	Amber
Femoral IABP	Red
ST 120 – 150	Amber
ECMO Femoral or subclavian	Red
ECMO Single bicaval	Amber
Pulmonary artery catheter *	Amber
Shock of any cause with lactate >4mmol/L *	Amber
DVT/PE *	Amber
Cardiac Ischemia *	Red
Known or suspected severe Aortic Stenosis *	Amber
**Neurological**	
RASS -2 or +2	Amber
RASS < -2	Red
RASS +2 or combative *	Red
Delirium +tve and able to follow simple commands*	Amber
Delirium +tve and not able to follow simple commands *	Amber
Active management of ICP with ICP not in desired range *	Red
ICP monitoring without active management of ICP *	Amber
Uncontrolled seizures*	Red
Vasospasm *	Amber
Craniectomy *	Amber
Open Lumber drain not clamped *	Red
Subgaleal drain *	Amber
Spinal Precautions *	Red
Acute Spinal Cord Injury *	Amber
Sub arachnoid Haemorrhage with unclipped aneurysm *	Amber
**Surgical**	
Unstabilised major fractures *	Red
Open Surgical Wounds *	Red
**Medical**	

Item	RAG
Known uncontrolled active Bleeding*	Red
Febrile with temperature exceeding maximum	Not Collected
Active Hypothermia	Not Collected
Femoral Sheath	Not Collected

* documented in reasons not mobilised
